# Using Machine Learning to Predict Antimicrobial Resistance―A Literature Review

**DOI:** 10.3390/antibiotics12030452

**Published:** 2023-02-24

**Authors:** Aikaterini Sakagianni, Christina Koufopoulou, Georgios Feretzakis, Dimitris Kalles, Vassilios S. Verykios, Pavlos Myrianthefs, Georgios Fildisis

**Affiliations:** 1 Intensive Care Unit, Sismanogleio General Hospital, 15126 Marousi, Greece; 2 1st Anesthesiology Department, Aretaieio Hospital, National and Kapodistrian University of Athens Medical School, 11528 Athens, Greece; 3 School of Science and Technology, Hellenic Open University, 26335 Patras, Greece; 4 Department of Quality Control, Research and Continuing Education, Sismanogleio General Hospital, 15126 Marousi, Greece; 5 Faculty of Nursing, School of Health Sciences, National and Kapodistrian University of Athens, 11527 Athens, Greece

**Keywords:** machine learning, artificial intelligence, antimicrobial resistance, AMR, antibiotic stewardship, clinical decision support tools

## Abstract

Machine learning (ML) algorithms are increasingly applied in medical research and in healthcare, gradually improving clinical practice. Among various applications of these novel methods, their usage in the combat against antimicrobial resistance (AMR) is one of the most crucial areas of interest, as increasing resistance to antibiotics and management of difficult-to-treat multidrug-resistant infections are significant challenges for most countries worldwide, with life-threatening consequences. As antibiotic efficacy and treatment options decrease, the need for implementation of multimodal antibiotic stewardship programs is of utmost importance in order to restrict antibiotic misuse and prevent further aggravation of the AMR problem. Both supervised and unsupervised machine learning tools have been successfully used to predict early antibiotic resistance, and thus support clinicians in selecting appropriate therapy. In this paper, we reviewed the existing literature on machine learning and artificial intelligence (AI) in general in conjunction with antimicrobial resistance prediction. This is a narrative review, where we discuss the applications of ML methods in the field of AMR and their value as a complementary tool in the antibiotic stewardship practice, mainly from the clinician’s point of view.

## 1. Introduction

In recent years, increasing antimicrobial resistance (AMR) has become a serious global concern. After having been exposed to antibiotics, bacteria can quickly develop resistance due to their short growth cycle and various adaptation mechanisms.

Multidrug-resistant, extensively resistant, or even pan-drug-resistant microorganisms are commonly encountered in a hospital environment, especially in the critical care setting [1,2]. According to estimates from the European Union/European Economic Area (EU/EEA), every year, more than 670,000 infections are caused by bacteria resistant to antibiotics, resulting in approximately 33,000 deaths [3]. The WHO European Region continues to experience high levels of antimicrobial resistance, particularly in the southern and eastern parts. The increasing resistance of *Klebsiella pneumoniae* and *Acinetobacter* spp. to third-generation cephalosporins and carbapenems and the rapid dissemination of resistant isolates limit antimicrobial choices for patients infected with these pathogens [2,4]. Failure to recognize a patient’s risk factors for infection with an antibiotic-resistant pathogen is the root cause leading to inappropriate treatment and eventually to unfavorable outcomes [5]. Several factors slow the development of new antibiotics, including a shortage of government research resources due to the financial crisis and extensive regulatory procedures for new drugs. More importantly, antibiotic development is no longer considered an economically viable investment in the pharmaceutical industry, because antibiotics are used for a relatively short period of time, unlike drugs used to treat chronic diseases. The cost of developing the latter is, first of all, much lower than that of antibiotics [6]. Therefore, over the past 15 years, there has been a significant gap in the development and availability of new antibiotics to address emerging resistance situations [4].

To deal with this rapidly growing problem, it is necessary to implement a multidisciplinary intervention known as an antimicrobial stewardship program (ASP) to optimize antibiotic prescription through evidence-based clinical decisions, and limit AMR [7]. Concerns are being expressed about the implications that the COVID-19 pandemic may have on antimicrobial stewardship programs and the further increase in AMR [8]. Overburdening of healthcare systems, increased workload of medical and nursing staff, and depletion of structural resources are some of the factors that potentially affect AMR hospital transmissions. Excessive and frequently unnecessary broad-spectrum empirical antibiotic prescribing in COVID-19hospitalized patients is commonly observed, exposing patients and the community to an increased risk of multidrug-resistant infections [9].

At the same time, the global increase in the use of electronic health records (EHR) has resulted in massive amounts of routinely available electronic patient and microbiological data that could be used to support individualized antimicrobial stewardship [10]. In previous years, a large proportion of clinical and laboratory data was disregarded or not collected at all. Several factors contributed to this limitation, including the size and complexity of the data, as well as the absence of techniques for collecting and storing them. Machine learning (ML), through complex processes, facilitates the optimal use of these data for evidence-based decision-making. From a large amount of clinical and laboratory data, machine learning can automatically extract meaningful rules, which, combined with the vast advances in computer processing power, are enabling the development of predictive tools [11].

Artificial intelligence (AI), through its ability to process data and information and turn it into insight and knowledge, facilitates data analysis that exceeds human mind capabilities and solves the problem of limited rational decision-making due to insufficient information and time constraints. AI, when properly designed, can also be free of behavioral constraints, including irrational deviations from guidelines, peer influence on hierarchical cultural norms, and fatigue. Algorithms can learn objectively and are often able to make more accurate predictions than those observed in everyday practice. In view of its potential promise in AMR, ML could greatly improve research efficiency, allowing scientists to focus on more complex scientific matters [12].

In this paper, we reviewed the existing literature on machine learning applications with respect to antimicrobial resistance prediction. This is a narrative review, where we discuss the applications of ML methods in the field of AMR and their value as an adjunct tool in the antibiotic stewardship practice, mainly from the clinician’s perspective. Recently, a systematic review of the literature with meta-analysis was published on the topic of ML-driven prediction of AMR [13]. In the current review, we have updated the literature search up to December 2022, and we principally emphasize the clinical context of ML applications, addressing healthcare professionals not quite familiar with AI technologies. Previous high-quality reviews exploring ML applications in the field of infectious disease mainly concentrate either on genomic-based technologies or on technical features of ML algorithms [11,14,15,16].

## 2. Materials and Methods

### 2.1. Search Strategy and Selection Criteria

**Figure 1:**
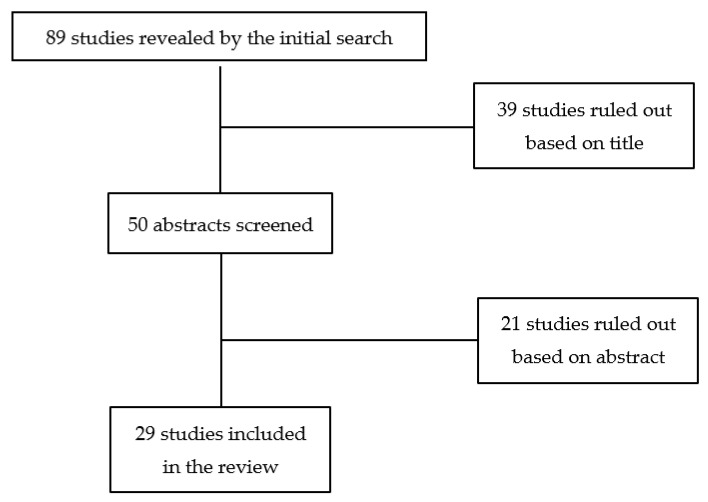
Flow chart of the included studies.

We investigated Medline/Pubmed up to December 2022 for original studies containing the keywords ((machine learning) AND (antimicrobial) AND (resistance) AND (hospital)) NOT (DNA) NOT (sequencing) NOT (peptides) NOT (molecules) NOT (genome) NOT (discovery)). As seen in the flow diagram (Figure 1), our research initially revealed eighty-nine studies. Thirty-nine studies were excluded by title screening. By abstract screening, twenty-one studies were also excluded. In this review, we included prospective and retrospective original studies in English that used ML algorithms to predict AMR in primary, secondary, and tertiary care, including intensive care, based on demographic, clinical, laboratory, and microbiological data to support clinical decisions. Investigations with ML systems that use genomic data (e.g., genome sequencing), as well as investigations aiming at the development of novel anti-infective agents through ML algorithms, were not included in this review. Twenty-nine studies exploring ML performance in AMR prediction were reviewed.

### 2.2. Popular Machine Learning (ML) Algorithms in the Field of AMR

Supervised machine learning algorithms are commonly used with linear and logistic regression, k-nearest neighbors (k-NN), support vector machine (SVM), decision tree (DT), random forest (RF), and gradient boosting machine (GBM), the most prevalent algorithms, followed by neural networks and deep learning approaches. The area under receiver operating curve characteristic (AUROC) is the main performance metric used in ML-driven prediction models. Other performance metrics include accuracy, sensitivity, specificity, positive and negative predictive values, precision, recall, and F1 measure, all of which, however, can be derived from a confusion matrix, a simple tabular structure that essentially summarizes the hits and misses of a learning algorithm.

## 3. Machine Learning (ML) Applications in the Field of AMR

There is growing evidence that, despite increased resistance to antibiotics, machine learning can help doctors select proper anti-infective treatment, based on patient demographics and past clinical histories [17]. During the past 20 years, the use of AI applications in several healthcare areas has increased significantly. As conventional computing methods are no longer suitable to handle and analyze big data, the exploitation of existing large medical databases by the use of ML has highlighted their value and, therefore, they have started to proliferate. Most machine learning (ML) systems in the field of antimicrobial resistance place an emphasis on research, medication development, or clinical microbiology [10].

More specifically, ML methods have been developed to analyze bacterial genomes, forecast medication susceptibility, recognize epidemic patterns for surveillance purposes, or propose new antibacterial treatments or vaccines [1]. In addition to developing new antibiotics, optimizing the use of current drugs has also been a key priority in stopping the spread of AMR, as one of the main drivers of AMR is the inappropriate use of antibiotics [4,5].

Several patient characteristics including comorbidities, demographics, previous infection history, antibiotic treatments, and hospital admissions have been associated with multidrug-resistant (MDR) infections in a number of studies [18,19]. The identification of risk factors, however, does not necessarily translate into highly accurate predictions. Several studies have therefore focused on using machine learning to improve clinical decision-making and antibiotic selection, particularly in the context of choosing empirical therapy [20]. Machine learning methods are first trained on multiple patient records and antibiotic resistance measures. A trained outcome model can predict a resistance profile based on patient clinical and microbiological data, and the results are used to select the most appropriate antibiotic regimen to treat an infection. A separate group of patient data is used to assess the accuracy of this trained model, comparing predicted and observed resistance outcomes.

### 3.1. Diagnosis of AMR

Currently, AMR is principally diagnosed using two techniques in clinical microbiology [11]. One is classical culture-based antimicrobial susceptibility testing (AST), and the other is whole-genome sequencing for antimicrobial susceptibility testing (WGS-AST) [1]. Although the former approach is simpler and easier to use, it typically requires a day or more to produce the results, which significantly lengthens the empirical antibiotic regimen and raises the possibility of treatment failure due to ineffective therapy or the threat of antibiotic resistance caused by broad-spectrum antibiotics.

The implementation of ML methods has substantially reduced the time of bacterial susceptibility profiling to less than three hours for the flow-cytometry AST method (FAST) [21] and only 30 min for the infrared (IR) spectrometry [22]. While these ML-assisted diagnostics can accelerate antimicrobial susceptibility testing, they require costly infrastructure and expert personnel to be carried out.

Although matrix-assisted laser desorption/ionization coupled to time-of-flight mass spectrometry (MALDI-TOF MS) is widely recognized as a reference method for the rapid and inexpensive identification of microorganisms in routine laboratories, little attention has been paid to its ability to determine AMR. Some recent studies have evaluated its potential use in conjunction with machine learning to detect AMR in clinical pathogens. [23,24]. Some ML-based MALDI algorithms are available for micro-organism identification and are also FDA approved, as, for example, the MALDI Biotyper CA (MBT-CA) System (Bruker Daltonics Inc, Billerica, MA) that was approved by FDA in 2013 [24].

Kirby–Bauer disk-diffusion and microdilution antibiograms are recommended as reference methods by the European committee on antimicrobial susceptibility testing (EUCAST) and the Clinical and Laboratory Standards Institute (CLSI) for determining antimicrobial resistance [25]. Results are usually qualitative and classified into categories, i.e., susceptible or resistant, depending on the breakpoint calibrated by the EUCAST, or expressed as minimum inhibitory concentrations (MICs) [25].

Although these conventional methods are effective, they are cumbersome, time-consuming, and do not enable the rapid choice of an effective targeted anti-infective treatment [23]. As the results cannot be obtained sooner than 48 h after receiving a sample, prolonged use or overuse of broad-spectrum antibiotics may result. For some pathogens, an even longer incubation time (72 h or more) is required [25]. Hence, rapid, accurate, low-cost diagnostic tests are needed to optimize antimicrobial use and minimize the potential selective pressures.

Whole genome system (WGS)-based diagnostic approaches are being used to overcome these limitations, especially for viral infections and tuberculosis, where culture-based microbiological diagnostics are either not applicable or time-consuming [1]. WGS can potentially alleviate many of these concerns by offering the potential to predict AST results by identifying the presence or absence of resistance genes, as well as mutations in relevant genes, from which clinicians can infer the activity of antibiotic agents [26]. However, the integration of WGS diagnostics in routine antibiotic surveillance and daily clinical practice has several challenges, especially in limited resource settings. These methods are more expensive and more complex to implement than standard antibiotic susceptibility testing [1].

The development of molecular tests has significantly contributed to rapid diagnostic testing and timely identification of pathogens and antibiotic resistance patterns, but their high costs and limited availability prevent them from being widely used. In this context, ML-driven predictive models of antimicrobial resistance may serve as a bridge between specimen collection and results from molecular and genotypic susceptibility analysis, facilitating time-sensitive empirical antibiotic choices.

### 3.2. Prediction of AMR

Accurate prediction of resistance against different antibiotics is directly beneficialfrom the patient’s point of view, because it helps avoid treatment failures. Such a prediction could have additional long-term benefits, for example, enabling the use of more targeted antibiotics, decreasing the need to use multiple antibiotics to cure the same infection, and lowering the risk of onward transmission. Machine learning algorithms have the potential to help clinicians predict antimicrobial resistance.

Besides the detection of antimicrobial resistance phenotypes, different ML modeling tools have been applied by several researchers to predict antibiotic susceptibility patterns of pathogens, allowing for the selection of the most appropriate treatment. Goodman et al. used recursive partitioning to build a decision tree for the prediction of extended-spectrum β-lactamase (ESBL) production in *Escherichia coli* and *Klebsiella spp.* bacteremia based on patient epidemiological and microbiological data [27]. Sousa et al. performed a prospective study to validate the decision tree (DT) designed by Goodman et al. in a cohort of bacteremic patients in a region with a high prevalence of ESBL [28]. In contrast with the earlier study, all types and species of β-lactamase producing Gram-negative bacilli were included. After increasing the cut-off values of certain variables associated with resistant infections, a modified DT was obtained with significantly improved performance compared to the original one. An analogous method was used by Guillamet et al. for the prediction of resistance to piperacillin–tazobactam, cefepime, and meropenem in patients with Gram-negative bloodstream infection. In their study, a good overall agreement in accuracies between multivariable logistic regression models and clinical decision trees that were developed using a recursive partitioning algorithm (Chi-squared automatic interaction detection) was observed [29].

Moran et al. assessed the accuracy of an open-source machine learning algorithm (XGBoost), trained in predicting antibiotic resistance for three Gram-negative bacterial species isolated from patients’ blood and urine within 48 h of hospital admission [20]. The ML algorithm performed better than medical staff and a simple risk assessment tool. According to the authors, a point-of-care decision support system with real-time, tailored therapy recommendations based on interim diagnosis and patient risk factors has a specific role to play, although generalizability is limited as the algorithm was trained to predict resistance only in selected cases and specific antibiotics.

McGuire et al. demonstrated that clinical data retrieved from the EHR of the patients could be used to train an ML algorithm and predict the presence of carbapenem resistance at the time of culture collection [5]. The predictive model generated rather low sensitivity and positive predictive values (30%), but a high negative predictive value of 99% (AUROC 0.846). The authors suggest that although ML certainly cannot substitute rapid molecular testing, it may be able to empower the appropriate selection of antimicrobials in advance, in case of real-time integration into the EHR. Henderson et al. evaluated an ML classification model to discriminate possible predictors of MDR Enterobacterales infection in human immunodeficiency virus (HIV)-infected patients, who, compared to the general population, present increased vulnerability [30]. In the study population, the outcome of interest was rare, which restricted the performance of the classification algorithms as well as the number of predictors to be analyzed as a result. Garcia-Vidal et al. employed an ML approach with input data from the hospital EHRs in a cohort of hematological patients at the onset of febrile neutropenia to predict which patients would necessitate broad-spectrum coverage for multidrug-resistant Gram-negative bacteria (MDR-GNB), enabling a personalized antibiotic approach that allows to avoid antibiotics in case they are not necessary [31]. In a recent conference paper, it was suggested that a Random Forest classifier implemented in EHR data extracted by a feature selection (FS) process could provide early detection of MDR infections among intensive care unit (ICU) patients with an accuracy of 77% [32].

Recently, Feretzakis et al. evaluated five ML algorithms to identify predictors of antibiotic susceptibility using simple patient demographics, culture, and antibiotic susceptibility test results of patients being treated in medical wards [33]. The authors suggest that clinicians can make empirical treatment decisions based on insights gained from applying ML algorithms to local antimicrobial susceptibility data, with the best ML model achieving an accuracy of 75.8%. A low-cost method (which can be a simple database) that requires a basic microbiology Laboratory Information System and can be applied in ICUs, where AMR constitutes a serious threat, is proposed [34]. An ICU antimicrobial susceptibility dataset was used to evaluate a collection of very popular learning classifiers. Multilayer perceptron and J48 (C4.5) algorithms surpassed other models in terms of AUROC with values of 0.726 and 0.724, respectively. Further limiting the assessment to specific multidrug-resistant Gram-negative pathogens significantly increased the accuracy of ML models (AUROC 0.933), which could lead to an evidence-based clinical decision regarding empirical antibiotic selection [35]. The authors of a study conducted in a University Hospital in Spain propose a strategy based on feature selection and ML techniques to detect antimicrobial resistance to Pseudomonas [36]. They conclude that in clinical settings, ML algorithms such as LR, k-NN, DT, RF, and MLP can accelerate the workflow. The performance metrics of the ML algorithms are clearly reported in the aforementioned studies, but in most cases, ML algorithms’ performances were not evaluated against empirical clinical decisions made by physicians. Table 1 summarizes the performance of different machine learning algorithms for the prediction of AMR across examined studies.

### 3.3. Machine-Learning-Assisted Antibiotic Prescription

It is common for non-infection specialists to treat infections in hospitals. The physicians are encouraged to follow local antimicrobial guidelines and evidence-based policies. However, adherence to the prescribing policies tends to be deficient. Human and behavioral factors influence the doctor’s prescribing decisions. On the other hand, clinicians are frequently urged to overuse antibiotics due to worries about the high mortality linked to delayed prescribing in diseases such as sepsis, the increase in drug-resistant infections, and the lack of accurate diagnostics to enable dynamic decision-making [5,8]. In a cohort of over a thousand critically ill patients with Gram-negative bacteremia, a fourfold increase in mortality was attributed to the failure of administering an in vitro active antibiotic treatment within six hours of septic shock, emphasizing the need for timely and appropriate antibiotic treatment [7].

A strategy for combating inappropriate antibiotic prescriptions in community and nursing home-acquired urinary tract infections (UTIs) is described by Yelin et al. [37]. The resistance of cultured bacteria from UTIs to six commonly prescribed antibiotics was associated with a number of demographic factors, including a residency in a nursing home, alongside with history of UTIs and prior antibiotic prescriptions. Despite acknowledged biases in the study design, computer-driven drug recommendations seem to reduce the proportion of inappropriate prescriptions to 5%, compared with physicians prescribing inappropriately 9% of the time. A major drawback of the method is its inability to select the narrowest-spectrum antibiotic, among those with the lowest resistance. Similar approaches could be used to treat other bacterial infections when detailed patient data are available, as has recently been suggested for bloodstream infections in a hospital setting [38].

Kanjilal et al. applied a machine learning approach to hospital electronic health record data to predict the likelihood that first- and second-line antibiotics would be resistant to uncomplicated UTIs [39]. Using the algorithm, the least broad-spectrum antibiotic was recommended for each isolate. Compared to clinicians, the pipeline reduced both broad-spectrum and ineffective antibiotic prescriptions for UTIs in the cohort of patients, indicating clinical potential. In stone disease patients with UTI, Tzelves et al. described a method that uses readily available data from the Laboratory Information System and compared several classifiers following a tenfold cross-validation approach on two different versions of a single dataset; the first contained only information of Gram stain, while the second had knowledge of bacterial species [40]. The best classifier achieved an accuracy of 77% with only pathogen Gram stain known, and nearly 87% after identifying specific microorganisms. Using statistical learning techniques, a series of predictive models was developed by another group of researchers to estimate the probability of susceptibility to five commonly used antibiotics for in-hospital UTIs, with modest performance [41]. The goal of these studies is to provide insight into the proper selection of the right antibiotic. In comparison to retrospective clinician prescription, antibiotic prescribing policies supported by machine learning predictions have shown improved effectiveness [39].

However, most of these data-driven predictive models lack generalizability and should be retrained in different healthcare settings due to the dynamic nature of bacterial infections, differences in local susceptibility patterns, and the inconsistency of patient variables in electronic medical records [42]. With increasing antibiotic consumption, resistance patterns can change over time, requiring periodic retraining [4].

Another study analyzed the performance of antibiotic selections informed by personalized antibiograms in comparison with antibiotic selections made by clinicians; it also systematically evaluated the trade-off in performance when fewer broad-spectrum antibiotics are selected. By reducing the unnecessary use of broad-spectrum antibiotics that breed resistant organisms, empiric precision antibiotic prescribing with personalized antibiograms could improve patient safety and antibiotic stewardship [43]. In the study of Rich et al., the boosted logistic regression (BLR) models yielded the highest discriminative performance as compared to the decision tree (DT) and random forest (RF) models, yet the clinical decision support system developed in this study was moderately predictive of antibiotic-resistant UTIs (AUROC 0.57–0.66) [44]. Still, when resistance testing is not possible or not rapid enough, these models can inform decision-making.

Another major concern is the emergence of resistant infections despite susceptibility-matched treatment, often caused by a different strain than the original infection, as demonstrated by Stracy et al. [45]. Combining patients’ microbiome profiles and data on antibiotic use for urinary tract and wound infections, the researchers built an ML-driven algorithm for patient-specific recommendations to minimize antimicrobial resistance at the individual patient level.

In the aforementioned studies, algorithms and models have been developed for predicting antibiotic resistance based on epidemiologic factors. However, it remains largely unstudied whether they will affect antimicrobial prescribing when implemented into clinical practice [46]. Recently, a case-based-reasoning algorithm was incorporated into a hospital’s information system and was evaluated using real-world patient data to investigate the potential impact of the system on antibiotic prescribing practices. The algorithm provided appropriate antibiotic recommendations that were significantly narrower in spectrum compared to choices being made in current clinical practice by physicians [47]. Avoiding unnecessary antibiotic prescriptions is also of major importance for the promotion of antimicrobial stewardship. Wong et al. developed an ML-assisted mobile application to help inexperienced or busy Emergency Department doctors in Singapore decide whether to prescribe antibiotics for uncomplicated upper respiratory tract infections (URTIs) [48]. Likewise, in COVID-19 hospitalized patients, a supervised ML algorithm was successfully used to detect bacterial co-infections or secondary infections, thus supporting antibiotic prescribing decisions or recommending antibiotic discontinuation [49]. However, investigating ML applications to support diagnostic decisions is beyond the scope of this review.

Table 2 summarizes the performance of machine learning algorithms for antibiotic prescription assistance across different studies.

### 3.4. Machine Learning-Assisted Clinical Decision Support Systems (ML-CDSS)

As antibiotic resistance is a major cause of mortality, it is imperative that researchers develop rapid and efficient methods to guide the rational administration of antibiotics, collectively known as antimicrobial stewardship programs (ASPs) [3,7]. Antibiotic prescriptions can result in the selection of drug-resistant organisms, affecting not only individual patients but also a patient’s microbiome and society as a whole [4]. It is often difficult to make consistent decisions during infection management due to the dynamic nature of the situation. Integrating broad and complex information is essential to making responsible prescribing decisions [15,16]. Besides the evidence-based guidelines, there are several clinical decision support systems (CDSS) and biomarkers that are commonly used to guide treatment. In a recent review, various uses of machine learning for clinical decision support in infectious diseases were identified, including the support of diagnosis, the severity of disease prediction, and selection of appropriate antimicrobial treatment [14]. Currently used CDSS are computer-assisted expert systems, based on human expertise (knowledge-based), subsequently translated into rules that are manually programmed in the system, trying to simulate or reproduce the decision-making ability of an expert on a specific task [14,18,19]. In contrast to expert systems, ML-assisted CDSS are able to automatically learn and improve from data (data-based), define their own rules, and interpret unknown situations [14,15].

The development of ML-CDSS using minimum variables may be beneficial when data are not readily available across certain areas or when resources are limited [34]. Particular attention should be paid to which variables are used by the ML-CDSS to predict their outcome. Moreover, it is difficult to develop and validate ML-CDSS without high-quality clinical data. It is essential to build a comprehensive clinical database so that clinicians can use future machine learning tools with confidence.

Decision support models for empiric treatment of sepsis can integrate predictors of antibiotic resistance and permit rapid antibiotic de-escalation without endangering timely and sufficient treatment [50]. Moreover, previous antibiotic susceptibility results provide potent information to predict resistance to existing infections [51]. Sick-Samuels et al. constructed a decision tree by using recursive partitioning to predict the risk of broad-spectrum antibiotic (BSA) resistance in a cohort of septic pediatric patients based on five distinctive risk factors [52]. Nearly half of high-risk BSA-resistant episodes were incorrectly categorized as low-risk episodes, and 9% were incorrectly categorized as high-risk episodes. This could have resulted in either undertreatment or overtreatment, depending on the situation. An alternative approach could improve the sensitivity of the prediction algorithm by capturing additional patient characteristics or variables.

In a retrospective study carried out in a children’s hospital in Cambodia, Oonsivalai and colleagues, propose a patient-level data-driven decision support system using a variety of machine learning techniques [38]. Mainly targeting ceftriaxone, a third-generation cephalosporin, the most frequently prescribed empirical antibiotic in practice at their study site, they specifically concentrate on the value of using the predictive models to identify patients at high risk of being infected with organisms resistant to it. The age of the patient, an age-adjusted weight score, and whether the infection was acquired in the hospital or in the community were revealed to be the most crucial factors for predicting antibiotic susceptibility. These are objective variables that are frequently collected in most therapeutic settings. The models’ other variables can also be quickly and inexpensively gathered using brief questionnaires. The calculations that underlie the predictions can easily be carried out remotely using any Internet-connected device in a matter of seconds on a low-cost computer. This makes the strategy extremely suitable for settings in low- and middle-income countries (LMIC), which often have the largest illness burden and the most urgent issues with antibiotic resistance [45].

In a recent study, Liang et al. developed an ML-driven predictive model for the timely prediction of carbapenem-resistant (CR) GNB carriage among ICU patients within one week [53]. CR-GNB carriers can be predicted in real time, helping medical staff implement more targeted nosocomial prevention and control measures to avoid transmission. Probably the biggest highlight of the study is that it was prospectively validated for 4 months, which means that the model has been used in a clinical setting, having an accuracy of 84%. However, it is hard to determine whether developing the model is more effective than implementing other infection prevention tools.

Goodman et al. examined two methodologies for the development of clinical decision support tools: a conventional logistic regression-derived clinical risk score versus an ML-derived decision tree [54]. Although the performance metrics of the models for the prediction of ESBL bloodstream infection were comparable, the decision tree was more user-friendly, with fewer variables for the end user, whereas the risk score presented better discrimination and bigger flexibility for adjusting sensitivity and specificity.

Another group of researchers showed that deep neural networks outperformed multivariable logistic regression in predicting the generation of ESBL in community-onset Enterobacteriaceae bacteraemia. The large number of features used for training the model could be a potential drawback of this study [55]. For this reason, the authors suggest the integration of this deep neural network model into the electronic health system to facilitate automatic recovery and calculation of patient parameters. This would allow clinicians to make effective use of stored digital personalized health data, otherwise discarded in conventional linear statistical models.

Multivariate associations rule mining methods—a subset of unsupervised ML techniques originally used in market-basket analysis—may efficiently identify and quantify correlations between resistance patterns, enabling the identification and tracking of clinically relevant MDR through comparisons between relevant subsets of isolates. In the clinical context, the application of association rule mining in the antimicrobial susceptibility dataset could also offer better antibiotic treatment policies [56,57]. However, in the context of this study, we have not invested in reviewing unsupervised techniques, mainly because these tend to be associated with earlier phases of ML projects, where the application goal is still elusive and the data analysts are still searching for the exact nature of the problem to be solved; for the time being, this is less of an issue of the AMR domain, where assessing resistance is widely accepted as a clear goal.

Table 3 summarizes the performance of machine learning-assisted clinical decision support systems (ML-CDSS) across different studies.

### 3.5. Prediction of AMR in the Environment Employing AI/ML

The problem of AMR is multifactorial and arises from the interaction of bacterial evolution, human behavior, and environmental factors that play a significant role in the transmission of resistant bacteria and pathogen emergence [15]. There is no doubt that AMR has expanded considerably beyond strictly medical settings to include relevant aspects of the environment. Currently, there is a general consensus that intervention strategies should not be limited to consider only human and veterinary medicine, but that the environment should also be taken into account. Thus, an important challenge in AMR control is estimating the prevalence of antibiotic resistance genes (ARGs) in source environments. Furthermore, investigating the conditions and extent of environmental selection for resistance is critical to allow preventive measures [59].

Machine learning and deep learning models have been validated for the prediction of ARGs in various environmental sources, such as in recreational beaches, soil, wastewater, and in several geographical regions [60,61]. Jang et al. studied neural network techniques aiming to predict ARGs occurrence on beaches quickly and accurately, as well as to define the environmental variables that influence these predictions [62].

## 4. Discussion

Early detection of AMR remains challenging despite rapid diagnostic advances. A delay in diagnosis can prolong the period of ineffective antibiotic therapy. Statistical models for predicting drug resistance can play an important role, especially in settings where rapid diagnostic tests are unavailable or are difficult to perform due to a lack of resources.

While traditional regression techniques (linear/logistic) are long-established for the development of predictive models and risk scores, ML approaches based on complex patient data and medical information are increasingly gaining ground in clinical prediction, given their flexibility, practicality, and the ability to handle a large number of predictors [63]. However, in terms of validity and accuracy, there is no clear-cut evidence that ML-based prediction outperforms conventional statistical approaches. Christodoulou et al., in a previous systematic review, compared the performance of traditional statistical methods (e.g., logistic regression) with ML algorithms for the development of clinical prediction models with binary outcomes. In studies with a low risk of bias, the difference in the logit area under the ROC curve between LR and machine learning was 0.00 (95% CI −0.18 to 0.18) [64]. In the field of AMR prediction, the results from a recent meta-analysis were inconclusive regarding the performance benefit offered by ML algorithms compared to risk scores developed by classical statistical methods [13]. Depending on the situation and the type of predictive problem, ML algorithms may have advantages over traditional regression models.

There is often a lack of ML expertise among healthcare professionals that can make it difficult to construct and train a successful model, deploy it in production, and integrate it with the clinical workflow [65]. For healthcare professionals with limited ML knowledge, automated machine learning (AutoML) platforms may prove a valuable tool that can provide fast and reliable results [65]. AutoML, a developing field that seeks to automatically select, compose, and parametrize ML models in order to achieve the best performance on a given dataset, has emerged as a way to make ML techniques more comprehensible and user-friendly to non-experts [58]. However, as automation tools always attempt to hide some complexity from the end user, it should not be surprising that the default configurations of AutoML systems will not be suitable for all applications and, therefore, judicious usage goes hand in hand with a fundamental understanding of the underlying algorithms to avoid reaching erroneous conclusions. Ideally, by extracting actionable information from the data, selecting features, and summarizing the results, the involvement of data scientists can be important, especially during data preprocessing [58], but also during evaluation, and could also help with up-skilling medical professionals by making them increasingly confident to couple their everyday practice with ML assisted decision support.

Despite satisfactory results achieved by most studies using ML methods for the prediction of AMR, several issues exist that limit a wider ML adoption in clinical practice. Firstly, the lack of high-quality data is a prerequisite for successful machine learning modeling with accurate results. Data are usually obtained from electronic health records which are frequently unreliable due to entry errors, duplicate records, or missing observations; additionally, the cost of collecting, storing, curating, and analyzing data has to be taken into account for investing in departmental, institutional or wider-area infrastructures [66]. The second issue is the insufficient generalizability of many ML-based applications since they are only capable of processing datasets drawn from the same distribution, and this poses huge challenges for the AMR domain since any two hospitals are de facto different ecosystems. Transfer learning and few-shot learning are, therefore, likely to become increasingly relevant to future research in the field of AMR in order to make use of even limited data [15]. However, an additional real challenge stems from reality: the data in any given hospital context might also reflect the singularities of the demographics of the human population (for example, an industrial environment vs. an agricultural environment vs. an urban one). For that reason, one has to also consider the extent to which ML models suffer from biases (in the statistical sense) and whether such data flaws have to be tackled at the data curation level (for example, by sampling techniques, which could address discrimination concerns) or at the model level (by developing distinct models for different population strata, which could address effectiveness concerns). Thus, because of underlying ethical and medical issues, generalization of the findings of an interesting or promising experiment to the deployment of tools or methods which will be acceptable at the (medical) practitioner level, at the (hospital) administration level or at the (regulation) compliance level is a complex problem.

Data volume and quality are substantial determinants of machine learning performance. In a recently published systematic review, increased risk of bias due to retrospective methodology, nonhomogeneous data processing, and lack of external validation are suggested as potential reasons for the current limited clinical applicability of ML-based predictive algorithms [13]. In order for ML models to be adopted into daily routine, they must be externally validated on different datasets and have end-point outcomes evaluated in real-world studies or randomized controlled trials (RCTs) [67]. Essentially, this confirms that ML-based tools and methods for medical use do not exist in a vacuum and must be designed with a clear view of the targeted audience (the medical practitioner or the trained patient), respecting the well-established procedures that, to date, shape the way other medical discoveries eventually find their way to the market through regulation (for example, FDA has initiated the process of admitting ML-based solutions in a variety of settings, but AMR prediction is not yet one of them [68]).

## 5. Conclusions

Recently, there has been increased research interest in various ML applications in the field of antimicrobial resistance prediction with promising results. In this review, we examined the existing literature on the topic of AMR prediction using ML algorithms and their potential role in the antimicrobial stewardship practice through accurate prediction of multidrug resistance patterns, a more personalized antibiotic prescription, and the development of accessible clinical decision support systems.

It is undoubtedly true that in the future, AI will enhance our ability to support healthcare decision-making, but humans must still interpret information according to the unique circumstances of each patient. Clinical decision-making is complex and the utility of ML-driven approaches in real-world settings has to be proved before they can be integrated into the clinical workflow.

## Figures and Tables

**Table 1:** Performance of machine learning across different studies in predicting antibiotic susceptibility patterns.

Authors	Year of Publication	Medical Setting	Geographical Setting	Input Data	ML Algorithms	Performance Evaluation	Bacterial Species
Goodman et al. [27]	2016	Hospital admissions	USA	Blood cultures/AST	Recursive partitioning, DT	PPV 0.908-NPV 0.919	*Escherichia coli*, *Klebsiella pneumoniae*, *Klebsiella oxytoca*
Vazquez-Guillamet et al. [29]	2017	Hospital admissions	USA	EHR data/Blood cultures/AST	Recursive partitioning, DT	AUC 0.61–0.80	GNB
Sousa et al. [28]	2019	Hospital admissions	Spain	Clinical/demographic data/Blood cultures/AST	DT	AUC 0.76	BL-GNB
Moran et al. [20]	2020	Hospital admissions and primary care	UK	Blood/urine cultures	XGBoost	AUC 0.70	*Escherichia coli*, *Klebsiella pneumoniae* and *Pseudomonas aeruginosa*
Feretzakis et al. [33]	2020	Medical wards	Greece	Demographics/Cultures/AST/Bacterial Gram stain/Type of sample	MLR	AUC 0.758	All isolated bacterial species
Feretzakis et al. [34]	2020	Intensive Care Unit	Greece	Demographics/Cultures/AST/Bacterial Gram stain/Type of sample	LR, RF, k-NN, J48, MLP	AUC 0.726	All isolated bacterial species
Feretzakis et al. [35]	2021	Intensive Care Unit	Greece	Demographics/Cultures/AST/Bacterial Gram stain/Type of sample	JRip, RF, MLP, Class. Regr, REPTree	F-measure 0.884, AUC 0.933	*Pseudomonas aeruginosa*, *Acinetobacter baumannii*, *Klebsiella pneumoniae*
Martínez-Agüero et al. [36]	2019	Intensive Care Unit	Spain	Demographics/Clinical data/Type of sample/Cultures/AST	LR, k-NN, DT, RF, MLP	Accuracy for quinolone resistance 88.1 ± 1.6	*Pseudomonas, Strenotrophomonas*, *Enterococcus*
McGuire et al. [5]	2021	Hospital admissions	USA	Demographic, medication, vital sign, laboratory, billing code, procedure, culture, and sensitivity data (67 features)	XGBoost	AUC 0.846	Bacterial isolates with CR
Pascual-Sánchez et al. [32]	2021	Intensive Care Unit	Spain	EHR data	LR, DT, RF, XGBoost, MLP	AUC 0.76	MDR bacteria
Garcia-Vidal et al. [31]	2021	FN Hematological Patients	Spain	EHR data	RF, GBM, XGBoost, GLM	AUC 0.79	MDR-*Pseudomonas aeruginosa*/ESBL-E
Henderson et al. [30]	2022	HIV patients	USA	EHR data	PLR, naïve Bayes, gradient boosting, SVM, RF	AUC 0.70	MDR-E

EHR: electronic health record, AST: antibiotic susceptibility testing, DT: decision tree, PPV: positive predictive value, NPV: negative predictive value, RF: random forest, XGBoost: eXtreme Gradient Boosting, MLR: multinomial logistic regression, MLP: multilayer perceptron, JRip (RIPPER): repeated incremental pruning to produce error reduction, Class. Regr.: a classifier using regression, *k*-NN: k-Nearest Neighbors, GBM: gradient boosting machine, SVM: support vector machines, GLM: generalized linear model, AUROC: area under receiver operating curve, CR: carbapenem resistance, ESBL: extended-spectrum beta-lactamase, BL: beta-lactamase, GNB: Gram-negative bacteria, MDR-E: multi-drug-resistant Enterobacterales, PLR: penalized logistic regression, FN: febrile neutropenic, HIV: human immunodeficiency virus.

**Table 2:** Performance of machine learning-Assisted Antibiotic Prescription across different studies.

**Author**	**Year of Publication**	**Medical Setting**	**Geographical Setting**	**Input Data**	**ML Algorithms**	**Performance Evaluation**	**Bacterial Species**
Yelin et al. [37]	2019	Community and nursing-home	Israel	Demographics/Urine cultures/Past antibiotic prescriptions	LR and GBDT models	AUC 0.7 for amoxicillin-CA to 0.83 for ciprofloxacin	*E. coli*, *K. pneumoniae* and *P. mirabilis*
Hebert et al. [41]	2020	In-hospital patients	USA	Demographics/Urine cultures/Past antibiotic prescriptions	PLR	AUC 0.65 to 0.69	Bacterial isolates from urine cultures
Tzelves et al. [40]	2022	Emergency department/urology ward	Greece	Demographics/Gram stain/Bacterial species/Sample type/AST	MLR with a ridge estimator	AUC 0.768 (unknown bacteria) AUC 0.874 (known bacteria)	Bacterial isolates from urine cultures
Kanjilal et al. [39]	2020	In-hospital and outpatients	USA	Demographic/Urine cultures/Past antibiotic prescriptions	LR, DT, RF	AUC 0.56–0.64	Bacterial isolates from urine cultures
Lewin-Epstein et al. [42]	2021	In-hospital patients	USA	Electronic health record data/Antibiotic susceptibility results	LASSO logistic regression, NN, GBDT, ensemble ^†^	AUC 0.73–0.79 (unknown bacteria) AUC 0.8–0.88 (known bacteria)	Bacterial isolates from blood/urine/other cultures
Corbin et al. [43]	2022	Emergency Department	USA	Electronic health record data/Antibiotic susceptibility results	LASSO/Ridge logistic regressions, RF, and GBDT	AUC 0.64–0.74	Bacterial isolates from blood/urine/other cultures
Rawson et al. [47]	2021	Hospital admissions	United Kingdom	Clinical, microbiological, prescribing information	Case-Based Reasoning (CBR)	OR: 1.77; 95% CI: 1.212–2.588; *p* < 0.01	*Escherichia coli* bloodstream infections
Rich et al. [44]	2022	In-hospital and outpatients	USA	Demographics, previous diagnoses, prescriptions, and antibiotic susceptibility tests	DT, boosted logistic regression (BLR), RF	AUC 0.57–0.66	Bacterial isolates from urine cultures

GBDT Gradient Boosting Decision Trees, † Ensemble of all 3 algorithms, PLR: penalized logistic regression MLR: multinomial logistic regression NN: neural networks, DT: decision tree, RF: random forest, LR: logistic regression, URTI: upper respiratory tract infections, GNB: Gram-negative bacteria, BSIs: bloodstream infections, AUC: area under curve.

**Table 3:** Performance of machine learning-assisted clinical decision support systems (ML-CDSS) across different studies.

Author	Year of Publication	Medical Setting	Geographical Setting	Input Data	ML Algorithm	Performance Evaluation	Bacterial Species
Oonsivalai et al. [38]	2018	Hospital admissions	Cambodia	Clinical, demographic and living condition information	LR, DT, RF, Boost, SVM, k-NN	AUC: 0.74–0.85	Bacterial isolates in blood cultures
Elligsen et al. [50]	2021	Hospital admissions	Canada	Demographics, acquisition of bacteremia, previous hospital/ICU admission, AST, antibiotic prescriptions	LR models	Antibiotic de-escalation (29 vs. 21%; OR = 1.77; 95% CI, 1.09–2.87; *p* = 0.02)	GNB bloodstream infections
Sick-Samuels et al. [52]	2019	Pediatric hospital	USA	Demographic, clinical, and microbiological data	Recursive partitioning, DT	AUC 0,70	GNB BSIs
Cazer et al. [56]	2021	Hospital admissions	USA	Bacterial isolates, infection site, AST, resistance phenotypes	Association Mining	Average cLift: 5	*Staphylococcus aureus* isolates
Sakagianni et al. [57]	2022	Intensive Care Unit	Greece	Demographics/bacterial species/sample type/AST	Association Mining	Max Lift: 3.44	*Pseudomonas aeruginosa, Acinetobacter baumannii, Klebsiella pneumoniae*
Feretzakis et al. [58]	2021	Medical wards	Greece	Demographics/Gram stain/bacterial species/sample type/AST	Microsoft Azure AutoML (StackEnsemble, VotingEnsemble, MaxAbsScaler, LightGBM, SparseNormalizer, XGBoost)	AUC: 0.822	Bacterial isolates
Lee et al. [55]	2021	Hospital admissions	Hong Kong	Patient reference number/Date of culture/Bacterial species/Sample type/AST	Adaptive boosting, gradient boosting, RF, SVM, K-NN and NN *	AUC: 0.761	*Escherichia coli*, *Klebsiella spp.*, *Proteus mirabilis*
Liang et al. [53]	2022	Intensive Care Unit	China	Demographic data, vital signs, basic and primary diseases, important test indicators, operation histories and antibiotic use	RF, XGBoost, DT, multiple LR	AUC 0.78–0.91	*CR-GNB carriage*
Goodman et al. [54]	2019	Hospital admissions	USA	Blood cultures/AST	LR, DT	C-statistic LR:0.87 DT:0.77	ESBL bacteria

* SVM: support vector machine, NN: neural network, RF: random forest, LR: logistic regression, DT: decision tree, XGBoost: eXtreme gradient boosting, k-NN: k-nearest neighbours, eCSR: expected cross-support ratio, cLift: conditional lift, CR-GNB: carbapenem-resistant Gram-negative bacteria, ESBL: extended-spectrum beta-lactamase, AST: antimicrobial susceptibility test.

## Data Availability

Not applicable.
